# Effect of Eccentric Control Exercises on Patients with Frozen Shoulder and Mild to Moderate Disability: A Single-Group Pre-Post Study

**DOI:** 10.12688/f1000research.167369.1

**Published:** 2025-11-14

**Authors:** Jishnu Mohan MP, S.Rajasekar Sannasi, Glenisha Ancita Dsouza, Praveen Kumar

**Affiliations:** 1 Insitute of Physiotherapy, Srinivas University, Mangaluru, Karnataka, India; 2 Department of Physiotherapy, College of Health Sciences, Gulf Medical University, Ajman, United Arab Emirates

**Keywords:** frozen shoulder, eccentric control exercises, eccentric training, Shoulder condition

## Abstract

**Background:**

Frozen shoulder (FS) is a common musculoskeletal condition characterized by inflammatory contracture of the glenohumeral joint capsule, leading to restricted active and passive range of motion, particularly in external rotation. Eccentric control exercises have demonstrated effectiveness in managing various upper limb disorders, including subacromial impingement, tennis elbow, and rotator cuff tendinopathy. However, there is limited evidence on their efficacy in individuals with frozen shoulder. This study aimed to evaluate the effects of eccentric control exercises on pain, functional disability, range of motion, psychosocial outcomes, and patient satisfaction in individuals with FS and mild to moderate disability.

**Methods:**

A single-group pre-post design was used. Twenty patients with clinically diagnosed FS and mild to moderate disability participated. All underwent 20 sessions of supervised eccentric control exercises over four weeks. Outcome measures included the Shoulder Pain and Disability Index (SPADI), Numerical Pain Rating Scale (NPRS), shoulder range of motion (flexion, abduction, hand-behind-back, and external rotation), Tampa Scale of Kinesiophobia (TSK), and Pain Self-Efficacy Questionnaire (PSEQ). Assessments were conducted at baseline, post-intervention (4 weeks), and follow-ups at 3 and 6 months. A 6-point Likert scale was used to measure patient satisfaction post-intervention. Data were analyzed using Repeated Measures ANOVA.

**Results:**

All outcome measures showed statistically significant improvement post-intervention (p < 0.05), with the benefits maintained at the 3- and 6-month follow-ups. Effect size indices at 4 weeks demonstrated a large treatment effect across all variables, suggesting strong clinical relevance.

**Conclusions:**

Eccentric control exercises significantly improved pain, functional disability, range of motion, kinesiophobia, pain self-efficacy, and patient satisfaction in individuals with frozen shoulder and mild to moderate disability. These findings support the incorporation of eccentric training in rehabilitation programs for frozen shoulder.

## Introduction

Frozen shoulder (FS), also known as adhesive capsulitis, is a common upper extremity condition characterized by an inflammatory contracture of the glenohumeral joint capsule. This leads to progressive restriction of both active and passive shoulder movements, particularly external rotation. ^1^ The condition affects approximately 2–5% of the general population and is more prevalent in females between 40 and 60 years of age. ^2^ While pain is typically localized to the anterior shoulder, it can radiate to the anterolateral arm and significantly impair functional activities and quality of life. ^2, 3^

Despite substantial research, the pathophysiology of FS remains not fully understood. It is hypothesized to involve a nonspecific, chronic inflammatory response in the synovial tissue, resulting in thickening and fibrosis of the capsule, and subsequent limitation of joint movement. FS is also more frequently observed in individuals with comorbidities such as diabetes mellitus, cardiovascular disease, Parkinson’s disease, stroke, and Dupuytren’s contracture, as well as those with a history of neck or cardiac surgery, smoking, or hyperlipidemia. ^4^

The clinical presentation of FS varies with disease progression. In early, high-irritability phases, patients often report intense pain with minimal stiffness. As the condition advances, stiffness becomes the predominant symptom with reduced pain levels. ^3, 4^

Exercise therapy is widely regarded as an effective conservative management strategy for FS. ^5^ Early-stage interventions typically include pendulum (Codman’s) exercises, wall walks, pulley-assisted movements, and shoulder wheels. As pain subsides, rehabilitation progresses to stretching, isotonic exercises, rotator cuff and scapular strengthening, and joint mobilization techniques. ^6^

Eccentric exercises, those that involve muscle lengthening under load, have been found effective in treating various musculoskeletal disorders, including subacromial impingement syndrome, lateral epicondylitis, and rotator cuff tendinopathy. ^7– 10^ These exercises generate high mechanical tension, believed to promote remodelling of connective tissues and improve neuromuscular control. ^8^ Their metabolic efficiency and ability to induce tissue adaptation have made them a subject of growing interest in rehabilitation research.

Although eccentric training has demonstrated effectiveness in upper limb disorders, its application in frozen shoulder remains underexplored. Considering the potential for improving joint range, muscular flexibility, and tendon compliance, eccentric training may serve as a valuable therapeutic strategy in FS management.

Therefore, we hypothesized that eccentric control exercises targeting the rotator cuff and shoulder musculature would improve pain, range of motion, functional disability, fear of movement (kinesiophobia), and pain self-efficacy in patients with frozen shoulder and mild to moderate disability.

The aim of this study was to investigate the effectiveness of an eccentric control exercise protocol on physical and psychosocial outcomes in this population.

## Methodology

The study was conducted at an outpatient department in a medical college Hospital after obtaining approval from the authors affiliated institutions. This is a single group pre-post design which included 20 patients with FS with mild-moderate disability. The clinical trial registration number for the study is CTRI/2023/01/048754. The study was conducted from December 2022 to October 2023. The inclusion criteria were age between 40 and 65 years of both the gender, diagnosed case of frozen shoulder (equal limitation of active range of motion and passive range of motion and normal X ray) with mild to moderate disability <50% reduction of external rotation when comparing opposite side, for mild disability Pain intensity was 3/10 on numerical pain rating scale, No night pain or sleeping pain, both active and passive range of motion are equally limited, but can tolerate passive overpressure at end range of motion. For moderate disability, pain was 4-6/10 on numerical pain rating scale, periodic ache while sleeping or resting, both active and passive range of motion are equally limited, can tolerate basic shoulder loading. The Exclusion criteria include FS patients with calcifying tendinitis, Greater tuberosity fracture, Necrosis of the humeral head, Rotator cuff related shoulder pain, pseudo-frozen shoulder, Neoplasm, Osteonecrosis, Cervicogenic shoulder pain, Locked dislocation, Glenohumeral osteoarthritis.

### Outcome measures

The outcome measures include Shoulder Pain Arm and Disability Index (SPADI). ^11^ Tampa scale of Kinesiophobia (TSK), ^12^ Joint ROM using Mobile Inclinometer, ^13^ Numerical Pain Rating Scale (NPRS), ^14^ Pain self-efficacy questionnaire ^15^ and 6-point Likert’s pain satisfaction scale. ^16^

### Procedure

A total of 27 patients presenting with shoulder pain were screened for eligibility, of which 20 participants (10 males and 10 females) met the inclusion criteria and were enrolled in the final study. Written informed consent was obtained from all participants in accordance with the Declaration of Helsinki.

Prior to the intervention, a blinded outcome assessor measured the following parameters: shoulder joint abduction, internal rotation, external rotation, hand-behind-back (HBB) reach, the Shoulder Pain and Disability Index (SPADI), the Numerical Pain Rating Scale (NPRS), the Tampa Scale of Kinesiophobia (TSK), the Pain Self-Efficacy Questionnaire (PSEQ), and a 6-point Likert scale for pain satisfaction.

**Figure 1.**
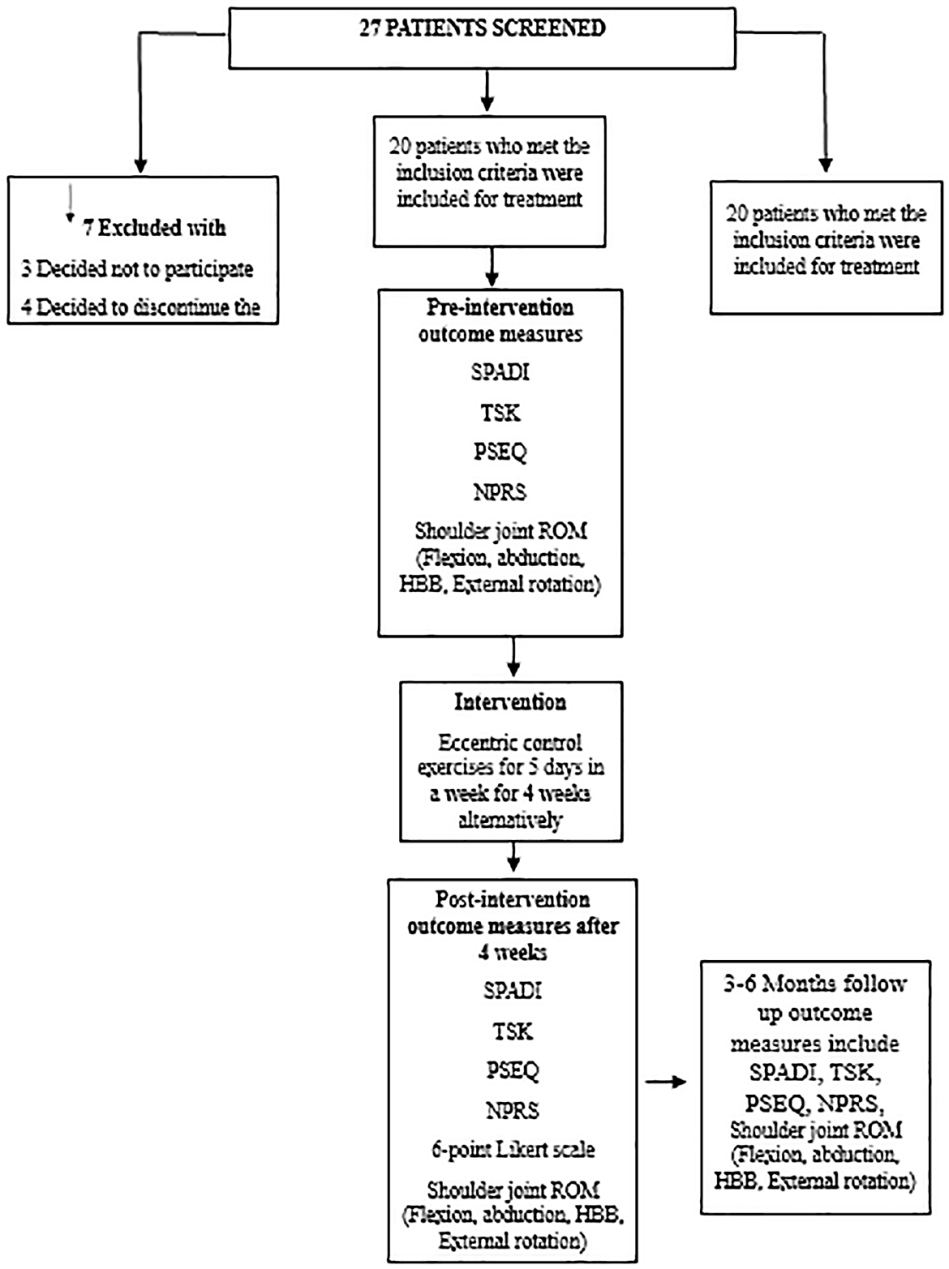
Flow diagram depicts the study outline.

The intervention consisted of eccentric control exercises, delivered every alternate day over a period of four weeks. Each session included three sets of 8–12 repetitions. Post-treatment outcomes were reassessed at the end of the 4-week intervention. The same eccentric exercise program was advised to be continued at home for follow-up assessments at 3 months and 6 months. The study outline is depicted in the flow diagram ( Figure 1).

**Figure 2.**
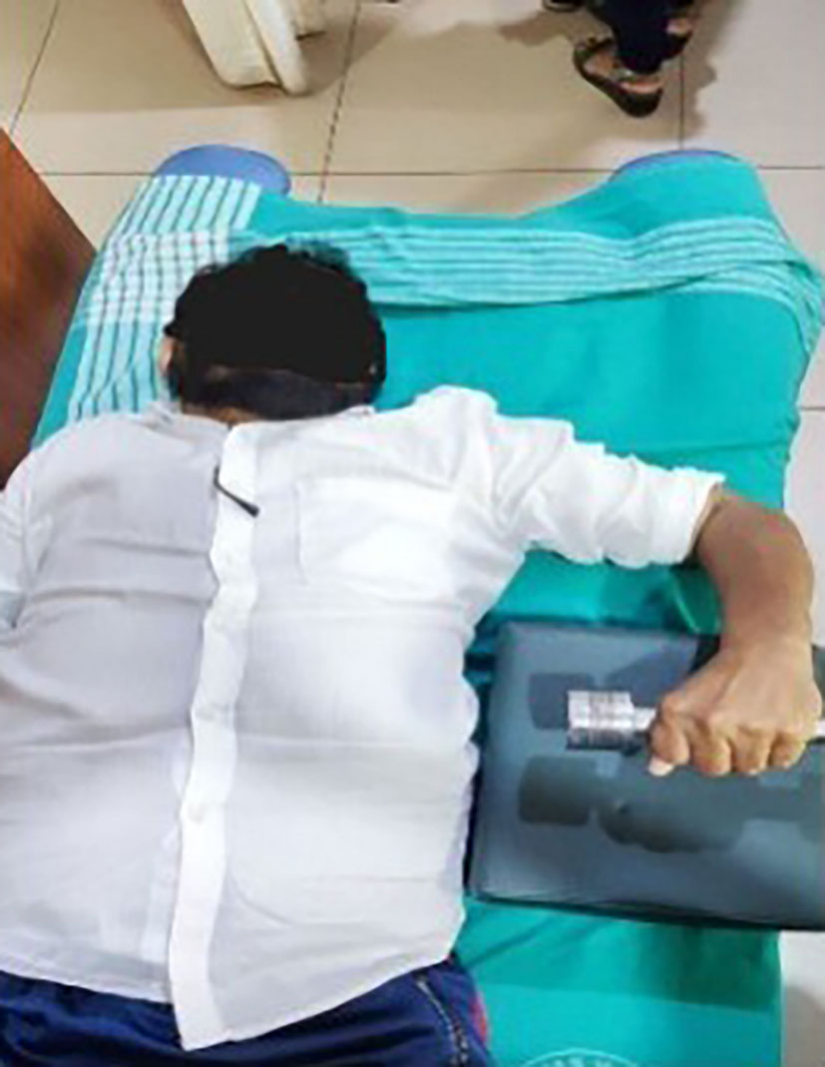
Eccentric exercise for external rotators.

**Figure 3.**
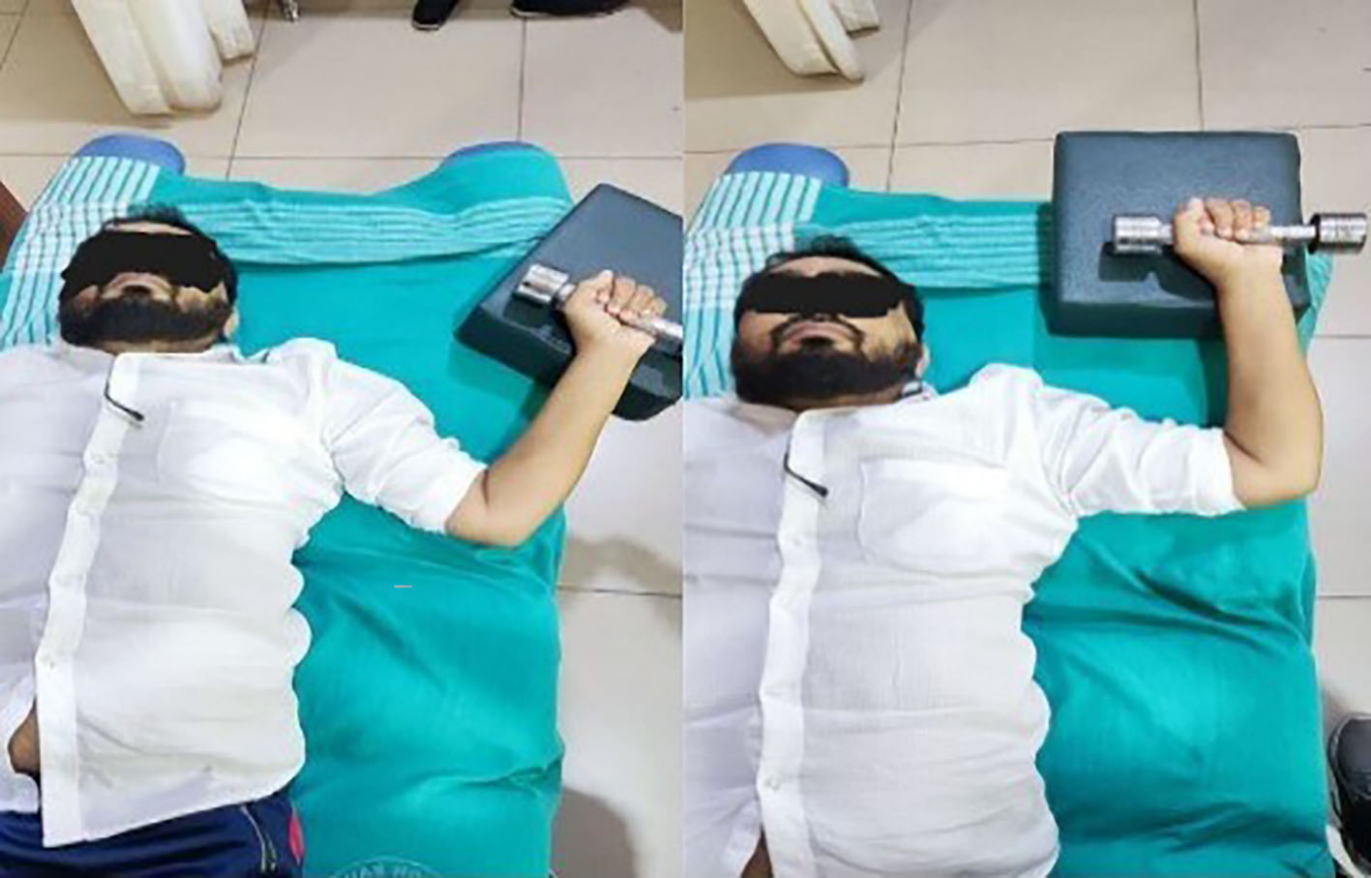
Eccentric strengthening for internal rotators (less abducted to more abducted position).

**Figure 4.**
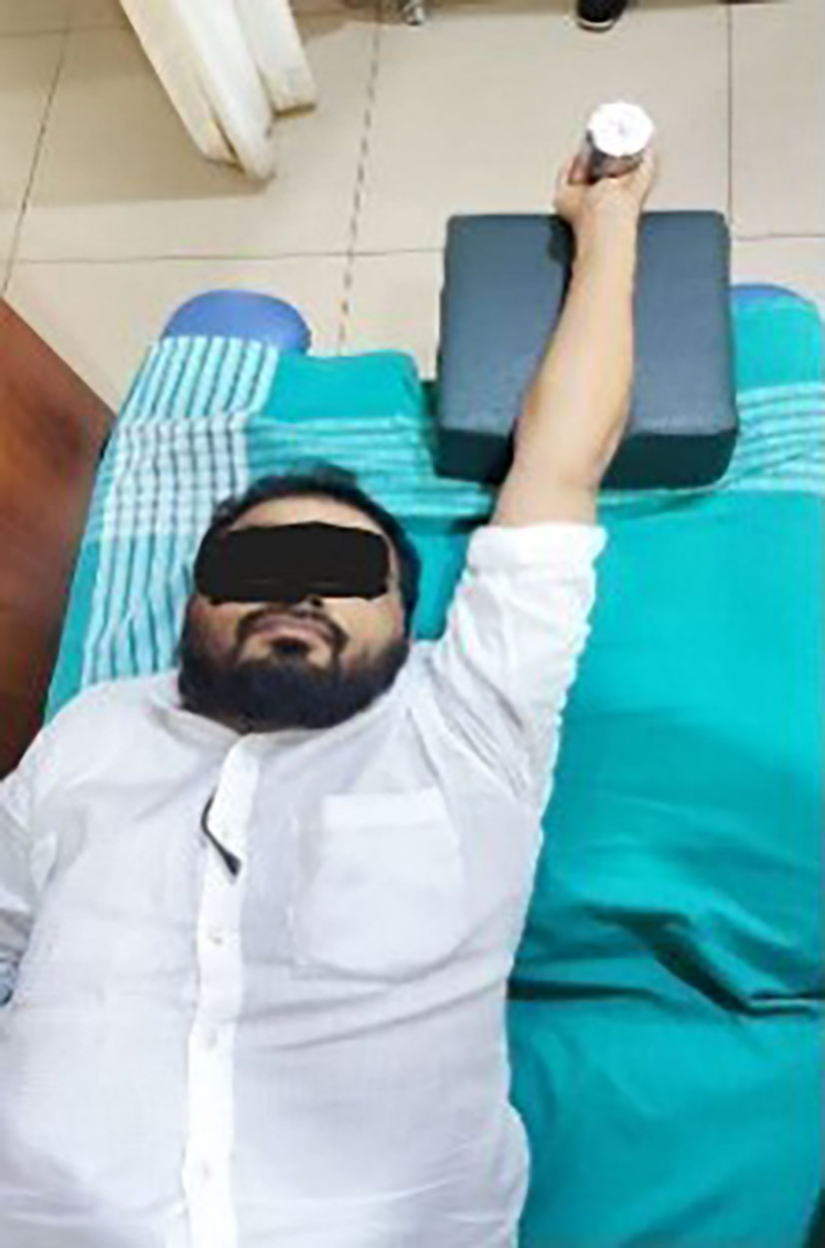
Eccentric exercise for flexors.

**Figure 5.**
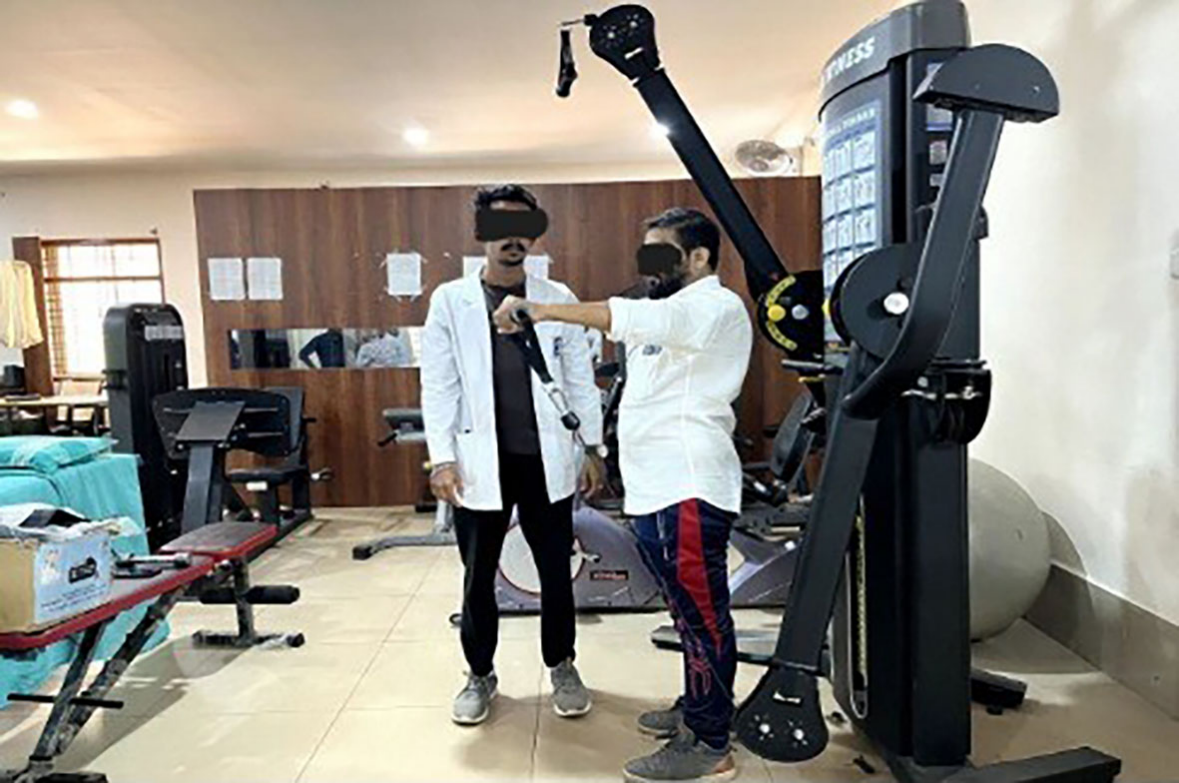
Progressive concentric and eccentric exercise for flexors.

**Figure 6.**
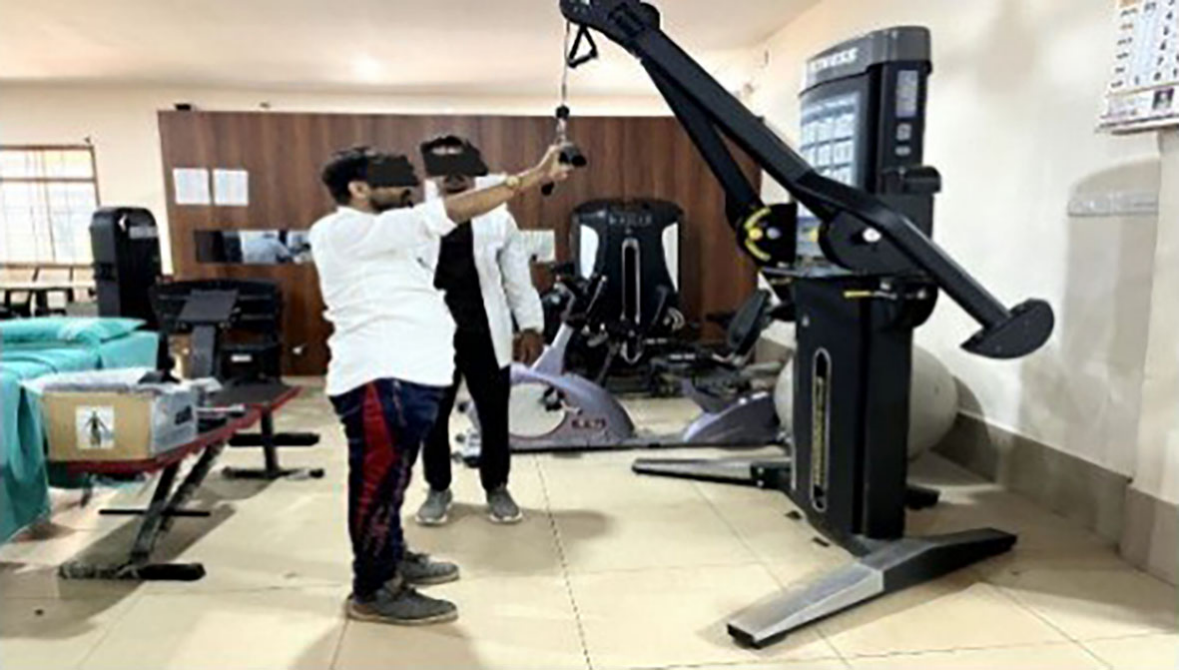
Progressive alternate concentric eccentric exercise for shoulder extensors starting position.

**Figure 7.**
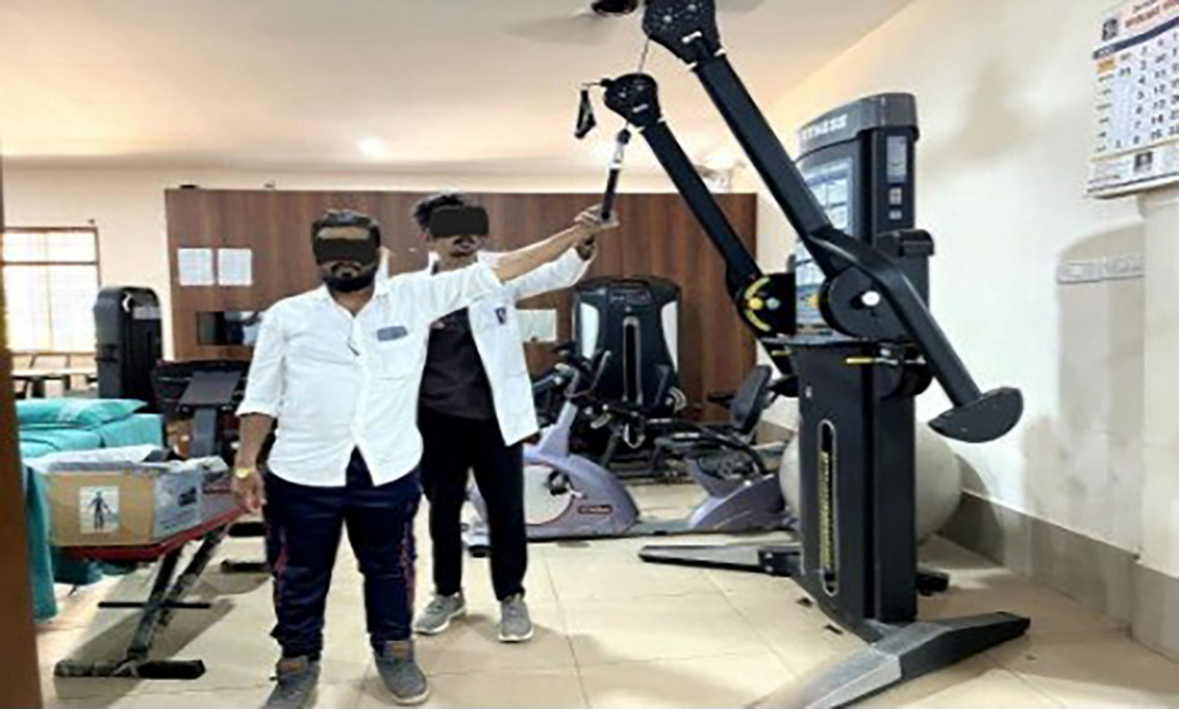
Progressive concentric and eccentric exercises for shoulder adductors.

**Eccentric exercises [ Figures 2- 7]**

External rotators: Participants were positioned in crook lying with the shoulder abducted and elbow flexed at 90°. They were instructed to perform active internal rotation using a 1 kg dumbbell, followed by passive external rotation performed by the therapist up to mid-range. During internal rotation, the external rotators undergo eccentric contraction. ^17^

Internal rotators: In crook lying, with the shoulder abducted and externally rotated, and the elbow flexed at 90°, participants performed controlled external rotation, followed by passive internal rotation by the therapist. The internal rotators contract eccentrically during the external rotation movement. ^17^

Flexors: Participants lay in a crook-lying position with the shoulder fully flexed. They were instructed to perform a controlled extension with a 1 kg dumbbell, followed by passive full shoulder flexion by the therapist. During the extension phase, the shoulder flexors undergo eccentric contraction. ^17^

Internal rotators with slight abduction: In crook lying with the shoulder slightly abducted and externally rotated, participants performed a controlled internal rotation using a 1 kg dumbbell. This was followed by passive external rotation by the therapist, maintaining the abducted position. This movement emphasizes eccentric contraction of the internal rotators. ^17^

### Ethical consideration and consent to participate

The study was conducted in adherence to the ethical principles outlined in the Declaration of Helsinki for research on human participant. Ethical approval to conduct the study was obtained from Srinivas University, Institutional Ethical Committee in August 20, 2022, with Reference number SUIP/PG22/114/2022. Written informed consent was obtained from each participant, ensuring their understanding and voluntary agreement to partake in the research.

### Data analysis

All statistical analyses were performed using the Statistical Package for the Social Sciences (IBM SPSS Statistics for Windows, Version 28.0). As the demographic data followed a normal distribution, results are presented as mean ± standard deviation (SD) along with the range. The effect size index was calculated for all outcome measures after the 4-week intervention to evaluate the magnitude of change. Apart from the Numerical Pain Rating Scale (NPRS), all outcome measures followed a normal distribution and were therefore analysed using repeated measures ANOVA to determine statistical significance across different time points. Since the NPRS data did not meet the assumption of normality, it was expressed as median with interquartile range (IQR) and analysed using the Friedman test to assess statistical significance over time.

## Results

The demographic characteristics of the recruited sample are presented in Table 1. Since the demographic variables follow a normal distribution, they are expressed as mean ± SD with range.

**Table 1.** Demographic dimensions of the sample recruited.

Demographic dimensions	Mean (SD)	Range
Age (Years)	55.9 ± 6.8	41 to 65
Height (cm)	161.9 ± 7.9	147 to 176
Weight (kg)	65.6 ± 9.9	51 to 83
BMI (kg/m ^2^)	24.9 ± 3.2	19.6 to 30

Table 2 displays the recorded data for outcome measures at baseline, post-intervention, 3-month follow-up, and 6-month follow-up.

**Table 2.** Outcome measures recorded at baseline, post-intervention, 3-month follow-up and 6-month follow-up.

Outcomes	Baseline	Post intervention	3-month follow-up	6-month follow up	p-value*
SPADI	80.5 ± 18.5	56.2 ± 12.7	28.7 ± 4.6	23.9 ± 2.9	<.001
TSK	36.8 ± 6.1	23.4 ± 4.1	18.4 ± 3.3	15.7 ± 2.8	<.001
PSEQ	42.6 ± 10.8	51.4 ± 4.0	54.9 ± 2.7	56.8 ± 1.5	<.001
NPRS	6.5 (6, 7)	4 (3.3, 5)	3 (2, 3)	1.5 (1, 2)	<.001 ^#^
SFl	144.1 ± 8.6	158.5 ± 5.0	167.6 ± 3.9	170.9 ± 2.9	<.001
SAb	129.9 ± 11.6	152.9 ± 7.6	165.4 ± 6.8	170.1 ± 4.2	<.001
SHbb	22.6 ± 4.8	35.9 ± 4.1	48.9 ± 5.7	53.1 ± 3.8	<.001
SEr	51.4 ± 9.6	61.9 ± 8.5	74.9 ± 5.7	81.6 ± 3.5	<.001

The effect size indices for the outcome measures SPADI, TSK, PSEQ, NPRS, SFI, Sab, SHb, and SEr were 1.48, 2.49, 0.93, 0.89, 1.93, 2.25, 2.96, and 1.15, respectively, indicating that the treatment was effective.

**Figure 8.**
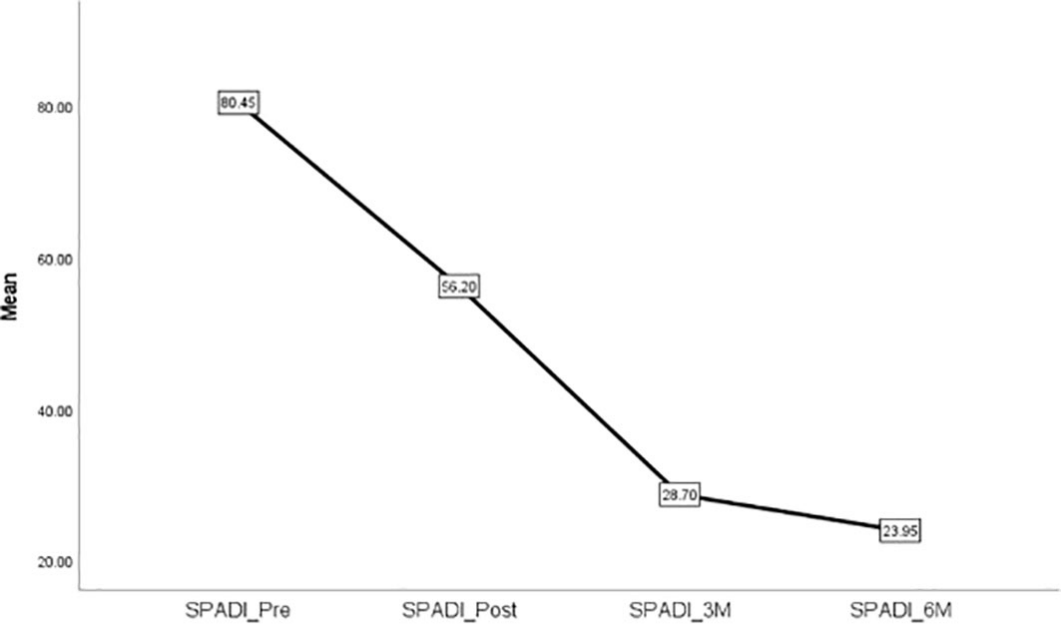
Timeline changes of SPADI in patients with FS.

Figure 8 illustrates the timeline changes in patients with frozen shoulder (FS).

**Figure 9.**
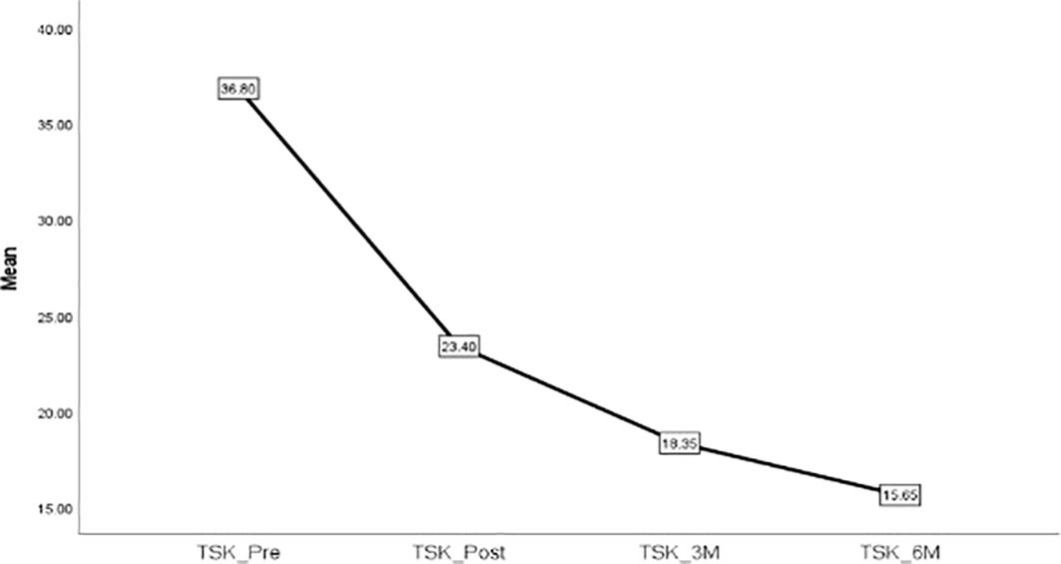
Timeline changes of TSK in patients with FS.

Figure 9 shows the timeline changes in TSK scores in patients with FS.

**Figure 10.**
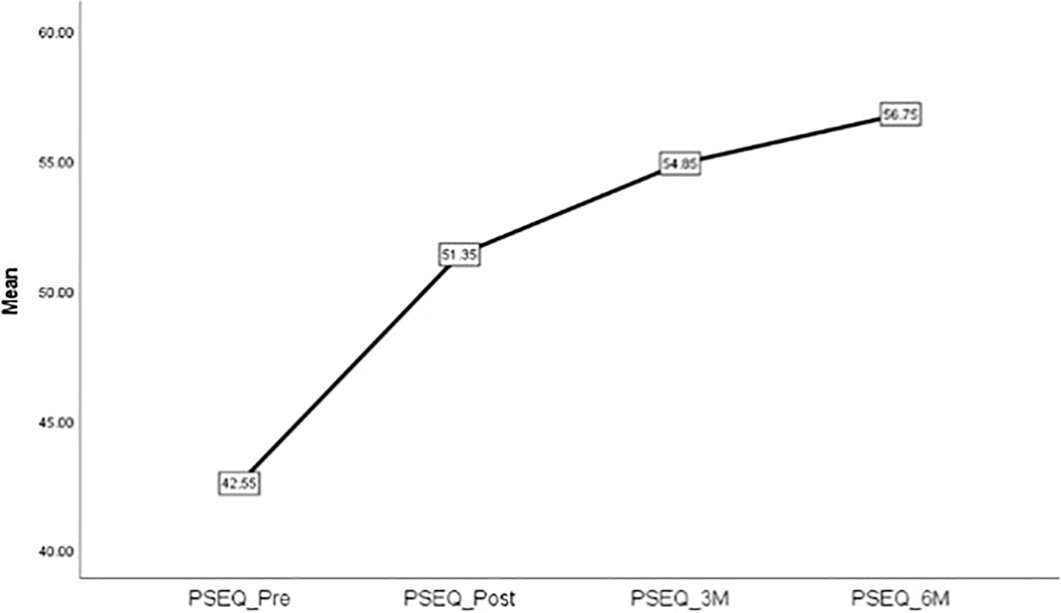
Timeline changes of PSEQ in patients with FS.

Figure 10 depicts the timeline changes in PSEQ scores in patients with FS.

## Discussion

This is the first study to evaluate the effects of eccentric control exercises in patients with frozen shoulder (FS) and mild to moderate disability, using a range of outcome measures including psychosocial parameters such as kinesiophobia and pain self-efficacy.

Previous research has shown that eccentric exercises outperform concentric exercises in improving pain, muscle strength, and function in various shoulder conditions such as subacromial impingement syndrome and rotator cuff tendinopathy. ^18– 20^ In the present study, the observed recovery in shoulder range of motion (ROM) may be attributed to an increase in sarcomere length and alterations in passive tension within the rotator cuff muscles or surrounding connective tissue structures. ^21^

Eccentric exercises have demonstrated rapid improvements in ROM, often with less energy expenditure than stretching. Unlike concentric contractions, which involve muscle shortening, eccentric contractions occur while the muscle lengthens under tension. This method of training is more metabolically efficient, as it requires less energy to produce the same force. ^22^ In addition to musculoskeletal benefits, eccentric exercises are known to enhance insulin sensitivity, promote muscle regeneration, improve lipid profiles, increase cortical excitability, and boost cardiorespiratory fitness. ^17^ Owing to these systemic effects, eccentric exercises have been utilized in managing conditions such as type 2 diabetes, sarcopenia, and cardiorespiratory disorders. ^17^

The primary goals of FS treatment include increasing both active and passive ROM, reducing pain, and improving shoulder function. ^23^ In our study, shoulder flexion, abduction, hand-behind-back (HBB) reach, and external rotation all showed notable improvements following the intervention. Importantly, our protocol involved a longer eccentric contraction duration of 10–15 seconds, which contrasts with earlier studies that adopted shorter durations. ^16, 17^

A systematic review concluded that eccentric training is more effective than concentric training for enhancing muscle mass in healthy individuals. ^18^ Additionally, individuals with subacromial pain syndrome demonstrated significant functional gains following eccentric exercise interventions. ^16^ Another study suggested that eccentric control exercises may be a key component of rehabilitation, particularly in female patients with FS. ^6, 18^ Based on these insights, we hypothesized that the improvements observed in our study may be due to mechanical changes such as reorganization of collagen fibers in the joint capsule and remodelling of adhered tissue. ^24^ Furthermore, increased synovial fluid circulation may have contributed to capsular tissue softening and increased joint mobilit. ^25^

Strength of the study: The internal validity of this study is strengthened by the standardized protocol, consistent outcome assessment tools, and blinded assessment of outcome measures. The study has several limitations that must be acknowledged. As a single-group pre-post design, it is vulnerable to threats such as history, maturation, and regression to the mean, which may confound the observed effects. The significant improvements seen across various timelines are encouraging, but without a control group, causal inferences should be made cautiously.

The external validity, or generalizability, is limited due to the small sample size (n=20), and single-centre design. While the results may be applicable to similar clinical populations in controlled settings, their extrapolation to broader populations (e.g., patients with severe FS, different age groups, or other comorbidities) should be done with caution.

**Suggestions for future research**

Future studies should consider using randomized controlled trials with larger and more diverse samples to compare eccentric exercises with other standard or emerging physiotherapy interventions. Inclusion of a placebo or active control group would help establish causal relationships. Additionally, further investigation into the neurophysiological and biomechanical mechanisms underlying the effects of eccentric loading in FS may enhance understanding and inform protocol optimization. It would also be beneficial to explore the long-term adherence to home-based eccentric programs and their impact on sustained functional recovery.

## Conclusion

Eccentric control exercises led to significant improvements in pain, functional disability, range of motion (including flexion, abduction, hand-behind-back, and external rotation), pain self-efficacy, kinesiophobia, and patient satisfaction in individuals with frozen shoulder and mild to moderate disability.

## Data Availability

Figshare: [data for eccentric exercise] https://doi.org/10.6084/m9.figshare.29491985.v2. ^26^ The project contains the following underlying data:
• Dataset for eccentric exercise Data are available under the terms of the Creative Commons Attribution 4.0 International license (CC-BY 4.0). Figshare: [data for eccentric exercise] https://doi.org/10.6084/m9.figshare.29491985.v2. ^26^ This project contains the following extended data:
• Screening form and data collection sheet • Informed consent Data are available under the terms of the Creative Commons Attribution 4.0 International license (CC-BY 4.0).
