# Supplementary material for: Effect of Eccentric Control Exercises on Patients with Frozen Shoulder and Mild to Moderate Disability: A Single-Group Pre-Post Study
Source: F1000Res. 2025 Nov 14;14:1257. [Version 1] doi: 10.12688/f1000research.167369.1 (PMC12809027; doi:10.12688/f1000research.167369.1)
Supplement: Peer review history

# Reviewer report: Reviewer response for version 1

**DOI:** 10.5256/f1000research.184479.r440018

**Published:** 2026-01-15

**Authors:** Ahmed M. El Melhat

**Affiliations:** 1 Cairo University, Giza, Egypt

Thank you for the opportunity to review the article entitled “Effect of Eccentric Control Exercises on Patients with Frozen Shoulder and Mild to Moderate Disability: A Single-Group Pre-Post Study”

 This study addresses an important rehabilitation question and presents encouraging pre–post improvements across pain, disability, ROM, and psychosocial outcomes following a short eccentric-oriented intervention for mild to moderate FS, with gains maintained up to 6 months.

 Kindly, find below the comments requiring responses/ justification from the authors.

**General Comment:**

 1.         Please clarify the exact mechanics of each “eccentric control” exercise: which muscle group was intended to be eccentrically loaded, what external torque was applied (direction and magnitude), how patients were instructed to resist, and the cadence/time-under-tension per repetition. Did you verify via EMG or force/torque proxies that the target muscle was active during lengthening?

 2.         There are inconsistencies in session frequency/dosage (20 sessions in 4 weeks vs every other day vs 5 days/week “alternatively”). What was the actual supervised schedule and total number of sessions per participant? How was home program adherence monitored during follow-up?

 3.         What was the predefined primary outcome, and was a sample size calculation performed? How did you handle multiple comparisons across outcomes and time points, and what effect size metrics were used for each test (please define and report CIs)?

 4.         ROM measurement details: Which inclinometer/smartphone app was used, what was the measurement protocol (positions, stabilization), and how was hand-behind-back quantified (units, landmarks)? you mean internal rotation of shoulder? Were intra-rater reliability checks performed for the blinded assessor?

 5.         Were any co-interventions (analgesics, injections, manual therapy, other exercises) allowed or recorded during the study and follow-ups? Please report adherence, adverse events (e.g., DOMS), and the missing satisfaction outcomes.

 7.         How do your results compare against standard-of-care interventions for FS (e.g., joint mobilization, corticosteroid injection plus exercise) in terms of magnitude of change and time course? Can you contextualize SPADI/NPRS changes relative to established MCIDs for clinical relevance?

 8.        Be consistent with case usage regarding the keywords.

 Methodology

 -I appreciate difficulty of recruiting this population, however, I am unsure about your criteria for inclusion, specifically had a corticosteroid injection or not. 

 -This is a very common treatment technique in these phases of frozen shoulder and by making this part of the exclusion really limits the clinical applicability of the study itself.  Also, how were patients diagnosed with frozen shoulder and by whom.  Additionally, what criteria were used to determine the phases?

 -You mention approval but do not name the ethics committee. plz Add approval number and institution.

 - Patient recruitment notes that inclusion criteria were participants mild to moderate disability, please elaborate on how this was defined/tested/operationalized

 -Several figures show machine-based cable exercises. Was this part of the standardized protocol? If so, please detail their purpose, dosage, and progression; if not, please adjust figures to match the Methods and avoid any crowded figures.

 -Flow diagram text appears truncated and inconsistent; attrition over 6 months is not reported.

 - Tables 2: Plz add all abbreviation below table.

 Discussion:

 The claim of being the “first study” in FS may be overstated given trials comparing contraction types in adhesive capsulitis; more nuanced positioning relative to **Kim et al. (2021)** and other FS-specific exercise literature is needed.

 -No comparisons to standard-of-care approaches for FS (e.g., joint mobilization programs, corticosteroid injections, combined manual therapy and exercise) to contextualize effect sizes. considered main limitation.

 Conclusions:

 -Satisfaction outcome is listed but not reported in Results despite being used in Conclusions.

Is the work clearly and accurately presented and does it cite the current literature?

Yes

If applicable, is the statistical analysis and its interpretation appropriate?

Yes

Are all the source data underlying the results available to ensure full reproducibility?

Yes

Is the study design appropriate and is the work technically sound?

Yes

Are the conclusions drawn adequately supported by the results?

Partly

Are sufficient details of methods and analysis provided to allow replication by others?

Yes

Reviewer Expertise:

Expert in musculoskeletal rehabilitation.

I confirm that I have read this submission and believe that I have an appropriate level of expertise to confirm that it is of an acceptable scientific standard, however I have significant reservations, as outlined above.

# Reviewer report: Reviewer response for version 1

**DOI:** 10.5256/f1000research.184479.r437368

**Published:** 2026-01-02

**Authors:** Indu Nanayakkara

**Affiliations:** 1 University of Peradeniya, Peradeniya, Central Province, Sri Lanka

The study purely looks at the effect of eccentric exercise affecting FS. It does not compare it with any other protocol or a control group. Knowing that FS has a natural course of action to become better over time (self limiting) we do not know whether the effect of the natural course of the disease is taken into account when these results are put forward. So I think when the authors  make conclusions, they have to take this fact into account.

 Other than that, the article is written well and can be indexed with making the above mentioned improvement to the discussion and conclusion.

Is the work clearly and accurately presented and does it cite the current literature?

Yes

If applicable, is the statistical analysis and its interpretation appropriate?

Yes

Are all the source data underlying the results available to ensure full reproducibility?

Yes

Is the study design appropriate and is the work technically sound?

Yes

Are the conclusions drawn adequately supported by the results?

Yes

Are sufficient details of methods and analysis provided to allow replication by others?

Yes

Reviewer Expertise:

Physiotherapy interventions, autonomic function testing

I confirm that I have read this submission and believe that I have an appropriate level of expertise to confirm that it is of an acceptable scientific standard.

# Reviewer report: Reviewer response for version 1

**DOI:** 10.5256/f1000research.184479.r440019

**Published:** 2025-12-30

**Authors:** Mohanakrishnan Jagadevan

**Affiliations:** 1 Jawaharlal Institute of Postgraduate Medical Education and Research, Puducherry, Puducherry, India

1) Pls mention why mild to moderate disability was selected

 2) Was the irritability assessed ,if so kindly highlight and justify the same

 30 Pls explain why abduction was not selected in your study for isolated eccentric protocol

 4) Was there any undesirable effects reported in this study

  5) In discussion "...................recovery in shoulder range of motion (ROM) may be attributed to an increase in sarcomere length and alterations in passive tension within the rotator cuff muscles or surrounding connective tissue structures." Pls explain how was this observed and pls justify your claim

 6) Kindly discuss the variability of the patient's response between mild and moderate disability and pain.

 7) Also adding a paragraph in discussion to describe the fear component in both the groups and the impact of eccentric exs on the same would add value to the paper

 8) Discussion has to be elaborated with your observations......

Is the work clearly and accurately presented and does it cite the current literature?

Yes

If applicable, is the statistical analysis and its interpretation appropriate?

I cannot comment. A qualified statistician is required.

Are all the source data underlying the results available to ensure full reproducibility?

Yes

Is the study design appropriate and is the work technically sound?

Yes

Are the conclusions drawn adequately supported by the results?

Yes

Are sufficient details of methods and analysis provided to allow replication by others?

Yes

Reviewer Expertise:

Shoulder, Knee , chronic pain and Yoga therapy

I confirm that I have read this submission and believe that I have an appropriate level of expertise to confirm that it is of an acceptable scientific standard, however I have significant reservations, as outlined above.
